# Notch Signaling Pathway Expression in the Skin of Leprosy Patients: Association With Skin and Neural Damage

**DOI:** 10.3389/fimmu.2020.00368

**Published:** 2020-03-19

**Authors:** Héctor Serrano-Coll, Juan Pablo Ospina, Lina Salazar-Peláez, Nora Cardona-Castro

**Affiliations:** ^1^Grupo de Ciencias Básicas, Doctorado en Ciencias de la Salud, Escuela de Graduados, Universidad CES, Medellín, Colombia; ^2^Línea de Investigación en Lepra, Instituto Colombiano de Medicina Tropical, Universidad CES, Medellín, Colombia; ^3^Laboratorio de Dermatopatología, Centro de Investigaciones en Dermatología (CIDERM), Facultad de Medicina, Universidad de Antioquia, Medellín, Colombia

**Keywords:** *Mycobacterium leprae*, Hes-1 transcription factor, Runx-1 transcription factor, inflammation, epidermis, hair follicle, eccrine gland, nerve fiber

## Abstract

**Introduction:** Leprosy is an infectious disease caused by *Mycobacterium leprae*, a debilitating disease that affects the skin and peripheral nerves. It is possible that tissue changes during infection with leprosy are related to alterations in the activity of the Notch signaling pathway, an innate signaling pathway in the physiology of the skin and peripheral nerves.

**Methods:** This is a descriptive observational study. Thirty skin biopsies from leprosy patients and 15 from individuals with no history of this disease were evaluated. In these samples, gene expressions of cellular components associated with the Notch signaling pathway, *Hes-1, Hey-1, Runx-1 Jagged-1, Notch-1*, and *Numb*, were evaluated using q-PCR, and protein expression was evaluated using immunohistochemistry of Runx-1 and Hes-1.

**Results:** Changes were observed in the transcription of Notch signaling pathway components; Hes-1 was downregulated and Runx-1 upregulated in the skin of infected patients. These results were confirmed by immunohistochemistry, where reduction of Hes-1 expression was found in the epidermis, eccrine glands, and hair follicles. Increased expression of Runx-1 was found in inflammatory cells in the dermis of infected patients; however, it is not related to tissue changes. With these results, a multivariate analysis was performed to determine the causes of transcription factor Hes-1 reduction. It was concluded that tissue inflammation was the main cause.

**Conclusions:** The tissue changes found in the skin of infected patients could be associated with a reduction in the expression of Hes-1, a situation that would promote the survival and proliferation of *M. leprae* in this tissue.

## Introduction

Leprosy is a neglected infectious disease caused by *Mycobacterium leprae*, an obligate intracellular microorganism that cannot be cultivated in axenic media (1). In addition, this mycobacterium is characterized by a marked affinity for the skin and peripheral nerve trunks and is responsible for the disability of more than four million people worldwide (2).

This disease has accompanied humanity for millennia, and it is a public health issue in some tropical countries, despite elimination efforts by the World Health Organization (WHO) (3). One of these efforts is the Global Strategy 2016–2020, which aims to reduce disability in children diagnosed with leprosy to zero and to reduce disability in new cases to less than one per million inhabitants (4). As published by Blok et al. (5), who used a simulated model to predict the future incidence of this disease in high-burden countries, it became clear that it is unlikely these WHO targets will be reached by 2020 unless additional measures are taken.

One measure that in the future could have an impact on reducing the burden and disability generated by *M. leprae* is to deepen our understanding of the physiopathogenic mechanisms related to disability and the proliferation of this mycobacterium in the host, from which tools can be generated to facilitate its detection and control. One of these mechanisms could be related to changes in the activation of the Notch signaling pathway, which is an innate cellular component involved in multiple processes of cellular proliferation and differentiation at the levels of the skin and peripheral nerves (6, 7).

In the nerve, our hypothesis is that Notch activation could be linked to the neural damage model proposed by Tapinos et al. (8), in which *M. leprae* induces the overexpression of cyclin D1 in the Schwann cell (SC), which generates dedifferentiation and demyelination of the nerve fiber. The association between cyclin D1 and Notch is plausible since, in other cellular models, it has been observed that cyclin D1 alone is not capable of generating changes at the cellular level. Indeed, changes are promoted through the activation of Notch (9) and the association of these two cellular components during infection. This indicates that the neural damage promoted by *M. leprae* could be linked to that reported by Woodhoo *et al*. (7), who observed that the postnatal activation of Notch induces the demyelination of the nerve fiber. Given that, the transcription factors of the Hes family, Hey, and even Runx-1 behave as repressors of the genes in charge of maintaining myelin in the nerve (10).

In the skin, we propose that *M*. leprae-induced modulation of Notch signaling pathway components - including Notch-1 receptor, Jagged-1 ligand, transcription factors Hes-1, Hey-1, Runx-1, and the Numb protein - could compromise the differentiation of cutaneous immune cells (11) and stem cells and even promote the demyelination of nerve fibers (10), facilitating the survival and proliferation of *M. leprae* and the manifestation of disabilities in the host.

Therefore, the aim of this study was to establish the relationship between the expression of components of the Notch signaling pathway (Hes-1, Hey-1, Runx-1, Jagged-1, Notch-1, and Numb) with tissue changes in the skin and dermal nerve fibers of leprosy patients.

## Materials and Methods

### Type of Study

A descriptive observational study was conducted with cross-sectional analysis.

### Sample Collection

Thirty skin samples were obtained from patients with multibacillary leprosy (MB) and active skin lesions (erythematosus + sensory compromise). These were collected in the departments of Santander, Norte de Santander, Boyacá, and Antioquia. The inclusion criterion for leprosy patients was that they had a clinical diagnosis of MB leprosy confirmed by bacteriological or histopathological examination. In addition, 15 skin samples were collected from discarded tissue resulting from plastic surgery from volunteer individuals with no personal or family history of leprosy.

Prior to the collection of these tissues, all participants gave their informed, written consent, which was endorsed by the institutional ethics committee for human research at CES University (Act No. 101 of 3 March 2017).

### Gene Transcription Analysis

#### RNA Extraction and Real-Time PCR (qPCR)

Skin samples were stored in RNAlater (Cat. No. 76104, Qiagen) at −20°C until RNA extraction. Total RNA was extracted using the RNeasy Mini Kit (Cat. No. 74104, Qiagen) following the manufacturer's instructions, and was eluted in 30 μL RNase- and DNase-free water (12). RNA concentration and purity were determined by spectrophotometry using a Nanodrop 1000 (Nanodrop Technologies, Wilmington, USA) and stored at −80°C until reverse transcription was performed.

RNA samples were reverse transcribed to create cDNA using RT^2^ First Strand Kit (Cat. No. 330404, Qiagen), following the manufacturer's instructions (13). RT-PCR was performed with a C1000TM thermocycler (Bio-Rad, Hercules, Ca, USA.)

qPCR reactions were carried out on a Custom RT^2^ PCR array (Cat. No. 33171, Qiagen) for the following genes: *Hes-1, Hey-1, Runx-1, Jagged-1, Notch-1*, and *Numb*. To each well of this array, 20 μL master mix prepared for each skin sample was added: 11.5 μL RT^2^ SYBR Green ROX FAST master mix (Cat. No. 330622; Qiagen), 1.5 μL cDNA, and 10 μL RNase-and DNase-free water. The conditions of the qPCR included a heating step for the activation of the Taq polymerase for 10 min to 95°C, followed by 40 amplification cycles (15 s at 95°C, and 30 s at 60°C). A dissociation or melting curve from 70°C to 90°C, with temperature increments of 0.5°C, was then plotted to confirm the amplification of the gene of interest (13). All qPCR reactions were carried out in a Corbett Rotor-Gene® 6000 thermocycler (Qiagen, Valencia, Ca, USA.).

The Ct (cycle threshold) values were recorded in an Excel spreadsheet and loaded into a software program provided by the manufacturer (Qiagen) for data analysis. Ct values were classified (leprosy and non-leprosy) and standardized using Ct averages from three housekeeping genes (GAPDH, RPLP0, and ACTB). Changes in gene expression were analyzed using the 2 ^−ΔΔCt^ method (14).

### Histopathological Analysis

#### Tissue Preparation for Histopathological Study

A fragment of the skin samples was fixed in 10% formalin blocking solution and paraffinized for the purposes of histopathological and immunohistochemical (IHC) evaluation. For histopathological evaluation, the paraffinized tissue was cut into 4–5-μm-thick sections, stained with hematoxylin and eosin (H&E) stain, and examined to confirm whether the tissue corresponded to healthy or *M. leprae*-infected skin. Histopathological evaluations were conducted by an expert dermatopathologist.

### H&E Analysis of Tissue Sections

In H&E-stained tissue sections, the following histopathological patterns were evaluated: trophic changes in the epidermis, location and severity of the inflammatory infiltrate, presence of granulomas and necrosis, and periadnexal and subcutaneous cellular tissue involvement (15). The information was recorded in a format designed for this purpose. There is a description of H&E staining in Supplementary Methods.

### Immunohistochemical Stain (IHC)

Skin tissue sections of 4 μm were deparaffinized in an oven at 58°C overnight, followed by three 5-min xylol immersions. After deparaffinization, the tissue was rehydrated using isopropanol solution dehydrated in absolute alcohol. The tissue sections were then blocked in a 6% hydrogen peroxide solution for 5 min, and epitope recovery was induced by heat in a water bath at 98°C and was performed with EDTA 10 mM.

After the antigenic recovery, the primary antibodies anti-Hes-1 (Cat. No. ab119776, monoclonal, dilution 1:100, Abcam), anti-Runx-1 (Cat. No. ab35962, polyclonal, dilution 1:250, Abcam), anti-S-100 (Cat. No. Z0311, polyclonal, dilution 1:5600, Dako), anti-CD68 (Cat. M0876, monoclonal, dilution 1:600, Dako), and anti-cyclin D1 (Cat. EP12, Master diagnostics) were added to each tissue section for each IHC assay. Each antibody dilution was performed in 1% bovine serum albumin (BSA) in 1X TBS (Table S1) then incubated for 1 h at room temperature. The primary antibody signal was then amplified with Quanto Amplifier (Cat. TL-125-QHL Thermo Fisher Scientific) for 10 min. After this, the secondary antibody—which is a specific polymer for anti-mouse and anti-rabbit IgG from the UltraVision™ Quanto Detection System HRP DAB (Cat. No. TL-125-QHL Thermo Fisher Scientific)—was incubated for 10 min according to the manufacturer's instructions. Staining patterns were developed using a 3.3' diaminobenzidine (DAB) solution for 3 min. Tissue sections were contrasted with hematoxylin, clarified with ammoniacal alcohol, and hydrated in pure isopropanol. As a negative control, the primary antibody was replaced by 1% BSA Figure S1. In addition, positive controls are reported in Table S1 and can be viewed in Figure S2. Finally, the specificity of the primary antibodies related to the Notch signaling pathway used in this investigation was confirmed through Western blot Figure S3 and Supplementary Methods).

### Immunohistochemical Analysis

The stained tissue sections were evaluated using two types of analysis, as follows.

Morphometric analysis was used in the regions of interest lacking inflammatory cell infiltrates. Briefly, the digital images were acquired using an optical microscope (Nikon Eclipse E200), captured under 40x magnification with a Lumenera (Infinity1) camera and stored in a TIFF (Tagged Image File Format) graphic format. Subsequently, 135 images were transferred to the free ImageJ version 1.52p software (NIH, Bethesda, Maryland), each having 2048 × 1536 pixels. In this software, the images were processed using the tool “Color Deconvolution,” which is an add-on that allows the breakdown of RGB color images and separates them into three complementary images (16). The first image corresponds to contrast staining with hematoxylin (R 0.65, G 0.704, B 0.286). The second image corresponds to the stain performed with the chromogen-DAB (R 0.268, G 0.570, B 0.776), and the third is a residual type image (R 0.711, G 0.423, B 0.561).

The supplementary images, corresponding to the staining with hematoxylin and DAB, were later converted to grayscale (8 bits) to create binary images possessing two pixel values, 255 pixels (white) and zero pixels (black) (17). Using the “threshold” command, an average threshold of 100 pixels was established on a histogram expanded for white color and zero pixels for black color in each of the evaluated images, which allowed for proper identification of the nuclei stained with DAB and hematoxylin. Finally, the command “analyze particles,” which scans the threshold image and measures the cells covered in accordance with the predefined parameters, was selected. In this way, the total number of cells stained with DAB and hematoxylin and the percentage of chromogen staining were determined. To validate the results of the morphometric analysis, a visual analysis in the areas of interest was carried out by an expert dermatopathologist, who was not involved with the ImageJ evaluation Tables S2, S3.

Visual analysis (semi-quantitative) was conducted by an expert dermatopathologist, using a visual score and carried out on skin structures compromised by inflammatory infiltrate. It is relevant to mention that this visual measurement was intended to reduce any information bias that could be generated in the digital analysis of these infiltrates into the tissue. Briefly, visual analysis was performed with an optical microscope (Leica dm500), using a magnification of 10x to 40x. This analysis was conducted under the guidelines of the College of American Pathologists, and the parameter chosen for this evaluation was the percentage of cells positive for each of the markers (18, 19). The visual score for the percentage of positive cells is, 0 ≤ 1%, 1 = 1–25%, 2 = 25–75%, 3 ≥ 75%.

The staining of skin nerve fibers marked with anti-S-100 was classified using the pathological patterns described in Table S4.

### Bacillary Index (BI) and IgM anti-NDO LID Serology

A description of these tests is available in the section Supplementary Methods.

### Statistical Analysis

The data were analyzed using the Statistical Package for the Social Sciences, version 21 (SPSS). The univariate analysis for qualitative variables was performed through the calculation of absolute and relative frequencies. In quantitative variables, measures of central tendencies (mean and median) were calculated. In addition, the normality of these variables was determined by the Shapiro-Wilk test. The bivariate analysis for qualitative variables was performed through Pearson's chi-square test, and the analysis of qualitative-quantitative variables was performed using the Mann-Whitney *U*-test or Student's *t*-test. The multivariate analysis was performed using a multinomial logistical analysis. The significance of the *p*-value was set to <0.05 for all analyses performed.

## Results

### Sociodemographic and Clinical Characteristics of Participants

Thirty patients with leprosy (90% male and 10% female), with a median age of 51 years, were evaluated. Using the WHO standard, 100% of the patients were classified as multibacillary, and 96.6% persisted with a positive BI. According to the Ripley-Jopling classification, 66.7% presented with lepromatous leprosy, 30% with borderline lepromatous leprosy, and 3.3% with borderline tuberculoid leprosy. In assessing the degree of disability, we found that 16.7% of the leprosy patients presented with Grade 0, 46.7% with Grade 1, and 36.6% with Grade 2; it is relevant to mention that 56.7% of patients had a history of having suffered from a type-II leprosy reaction, while the remaining 43.3% had not presented these immunological categories. In addition, 86.6% of patients were seropositive for IgM anti-NDO-LID (Table 1).

**Table 1:** Sociodemographic and clinical characteristic of the leprosy patients.

**Characteristic of the leprosy patients evaluated**		***n* = 30 (%)**
Sex	M	27 (90%)
	F	3 (10%)
Median age* (Range)		51 (28–65)
Geographic area	Santander (Norte y Sur)	20 (66.6%)
	Antioquia	4 (13.4%)
	Boyacá	6 (20%)
WHO classification	PB	0
	MB	30 (100%)
R&J classification	BT	1 (3.3%)
	BL	9 (30%)
	LL	20 (66.7%)
Grade disability	0	5 (16.7%)
	1	14 (46.7%)
	2	11 (36.6%)
History of leprosy reactions	No	13 (43.3%)
	LR1	0
	LR2	17 (56.7%)
Bacillary index	(+)	29 (96.6%)
	(-)	1 (3.4%)
IgM anti-NDO-LID	(+)	26 (86.6%)
	(-)	4 (13.4%)

With regard to the sociodemographic characteristics of the 15 non-leprosy individuals, 60% were female and 40% male, with an average age of 42.8 years, and none had any personal or family history of leprosy (Table S5).

### Histological and Histopathological Characteristics

Histopathological findings for leprosy skin: Atrophic changes were observed in the epidermis of 73.3% of patients. The skin of 96.7% of patients had some type of inflammatory infiltrate, and 76.7% presented granulomas.

At the level of the skin annexes, 83.3% showed alterations, 40% with infiltrate at the peri-eccrine level, 3.3% in the pilosebaceous unit, and 40% had mixed-type infiltrate (of the eccrine glands and in the pilosebaceous unit). With regard to subcutaneous cellular tissue, it was impaired in half of the leprosy patients, with a lobulillar-type impairment in 10% and mixed (lobulillar-septal) in 40%. It is also relevant to mention that areas of cutaneous necrosis were found in only 6.6% of patients. In addition, in the evaluation of dermal nerves of leprosy patients, trophic changes were observed in 96.7% of samples, showing absence in 50%, infiltrates in 43.4%, and fragmentation in 3.3% (Table 2).

**Table 2:** Histological and histopathological description.

**Histopathological and histological description**			
**Variables**		**Leprosy skin** ***n*** **=** **30 (%)**	**Non-leprosy skin** ***n*** **=** **15 (%)**
Epidermal atrophy	Yes	22 (73.3%)	0
	No	8 (26.7%)	15 (100%)
Epidermal Hyperplasia	Yes	3 (10%)	0
	No	27 (90%)	15 (100%)
Inflammatory infiltrate	No infiltrate	1 (3.3%)	15 (100%)
	Perivascular	15 (50%)	0
	Nodular	4 (13.3%)	0
	Diffuse	10 (33.4%)	0
Granuloma	Non-granuloma	7 (23.3%)	15 (100%)
	Epithelioid	13 (43.3%)	0
	Suppurative	10 (33.4%)	0
Necrosis	Yes	2 (6.6%)	0
	No	28 (93.4%)	15 (100%)
Periadnexal infiltrate	No infiltrate	5 (16.7%)	15 (100%)
	Pilosebaceous unit	1 (3.3%)	0
	Perieccrine	12 (40%)	0
	Mixed (pilosebaceous-perieccrine)	12 (40%)	0
Compromised subcutaneous cellular tissue	No compromise	15 (50%)	15 (100%)
	Lobular	10 (33.4%)	0
	Septal	0	0
	Mixed (lobular-septal)	5 (16.6%)	0
Morphology of dermal nerves	Intact	1 (3.3%)	15 (100%)
	Infiltrated	13 (43.4%)	0
	Fragmented	1 (3.3%)	0
	Absent/destroyed	15 (50%)	0

Histological findings for the skin samples of non-leprosy individuals: In all samples, no changes were observed, either trophic, inflammatory, or nervous, in the evaluated structures (Table 2).

When comparing the histopathological changes of leprosy and non-leprosy samples, a statistical difference was observed (Table S6).

### Differences in the Expression of Notch Signaling Pathway Components in Skin Samples of Leprosy Patients and Non-leprosy Individuals

**Figure 1:**
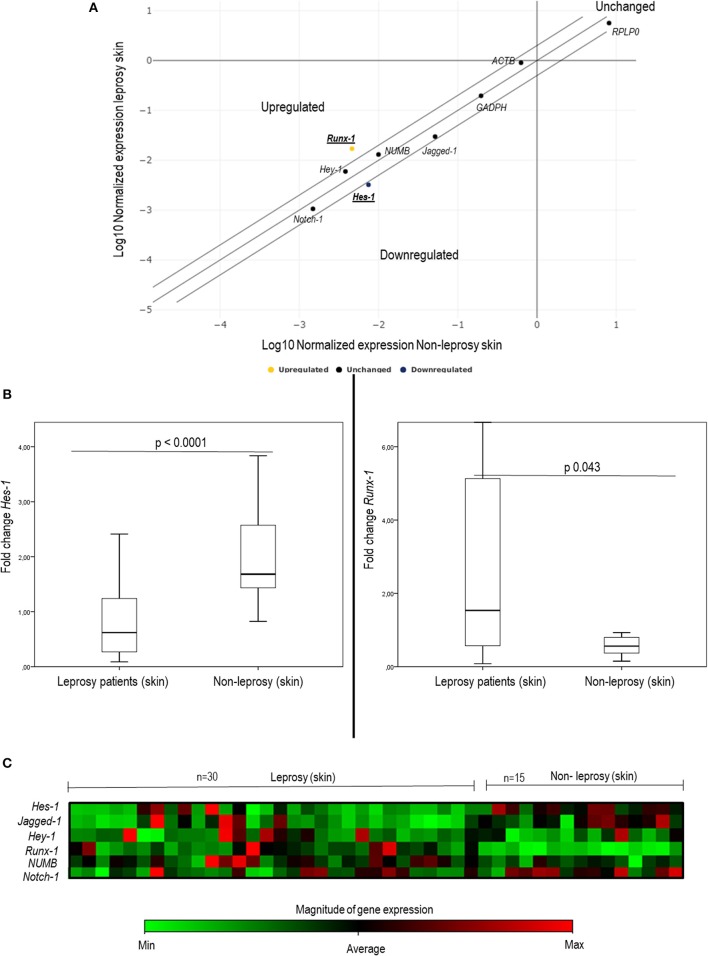
Changes in the expression of some components of the Notch signaling pathway in leprosy skin vs. non-leprosy skin using q-PCR. **(A)** The dispersion chart shows non-significative changes in genic expression (≤ 2) for *Notch-1, Jagged-1, Hey-1*, and *Numb* (black dots). Outside of the matrix, *Hes-1* and *Runx-1* show significant change (≥ 2), and there are significant changes in gene expression (≥2) for *Hes-1* (Blue dot - downregulated) and Runx-1 (yellow dot - upregulated). **(B)** Whisker box charts show lower expression of *Hes-1* and an increase in the expression of *Runx-1* in leprosy patients vs. non-leprosy (*p* < 0.05). **(C)** Heat map shows the gene expression of *Hes-1, Runx-1, Notch-1, Jagged-1, Hey-1*, and *Numb* in leprosy and non-leprosy patients. Green tones indicate a downregulated gene, and red tones indicate upregulated genes.

To establish whether there are changes in the expression of genes of interest (*Hes-1, Hey-1, Runx-1, Numb, Notch-1*, and *Jagged-1*), a dispersion chart was plotted. We found evident changes in the expression of two of the evaluated genes: *Hes-1*, downregulated 2.32 times, and *Runx-1*, upregulated 3.69 times in leprosy skin samples (Table 3, Figure 1A).

**Table 3:** Changes in the expression of some components of the Notch signaling pathway in leprosy skin vs. non-leprosy skin.

**Gen**	**Upregulated or Downregulated**	**U Mann-Whitney *p*-value**
***Hes-1***	**-2.32**	**<0.0001**
***Runx-1***	**3.69**	**0.043**
*Jagged-1*	−1.73	**0.009**
*Notch-1*	−1.40	0.08
*Hey-1*	1.56	0.79
*Numb*	1.31	0.67

*The bold values are showing statistical significance*.

When comparing gene expression quantification via the delta-delta Ct method, significant changes were noted in the expression of *Hes-1* (*p* < 0.0001), *Runx-1* (*p* = 0.043), and *Jagged-1* (*p* = 0.009) (Table 3, Figure 1B, and Figure S4). To confirm these changes and to characterize this difference in a clearer manner, a heat map was made, which provided a graphical and coded representation of colors related to the expression of these genes (green = reduced expression, red = increased expression). *Hes-1* was found to have reduced expression in 83.3% of the leprosy samples and increased expression in 67% of non-leprosy samples. *Runx-1* expression was increased in 33.3% of leprosy skin samples and in 100% of non-leprosy samples. *Jagged-1* was reduced in 83.3% of leprosy samples and increased in 53.3% of non-leprosy samples (Figure 1C).

### Identification of Runx-1 Expression in Cutaneous Structures of Leprosy Patients and Non-leprosy Individuals

**Figure 2:**
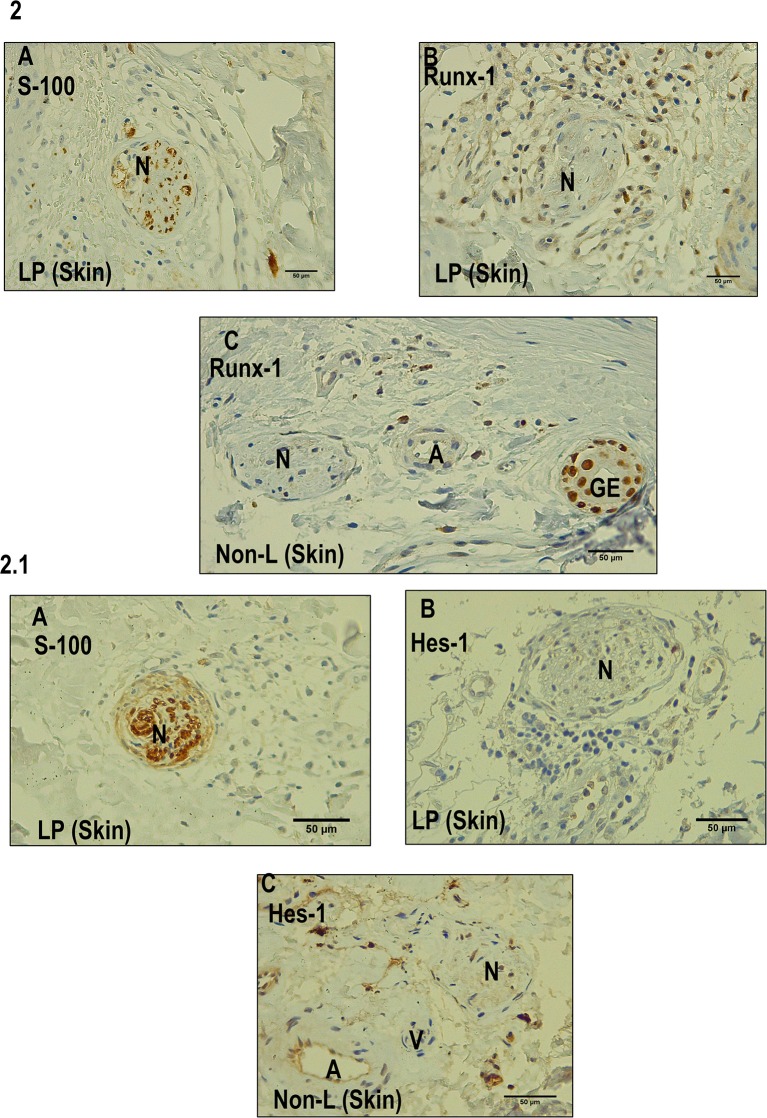
Immunohistochemistry with Runx-1 and Hes-1 in dermal nerves (leprosy skin and non-leprosy skin). **(A)** Dermal nerve (N) stained with S-100 in a leprosy sample (LP). **(B)** Dermal nerve (N) in a leprosy patient (LP) negative for Runx-1. **(C)** Dermal nerve (N) 1 in a non-leprosy sample (non-LP) negative for Runx-1. A: artery, EG: eccrine glands. All the figures have a 50-μm scale bar. **2.1 (A)** A dermal nerve (N) stained with S-100 in a leprosy sample (LP). **(B)** Dermal nerve (N) in leprosy sample (LP) negative for Hes-1. **(C)** Dermal nerve in a sample non-leprosy (Non-LP) negative for Hes-1. A: artery, V: venule, N: nerve. All the figures have a 50-μm scale bar.

**Figure 3:**
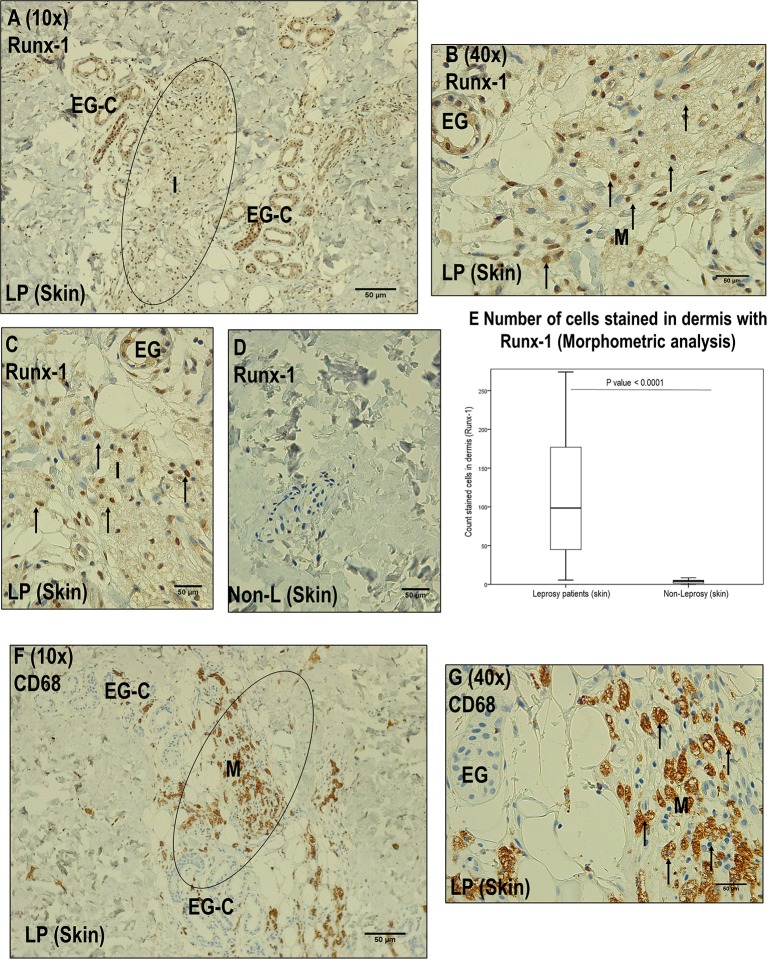
Immunohistochemistry with Runx-1 in leprosy skin vs. non-leprosy skin**. (A)** Image (10x magnification) showing leprosy dermis (LP), with inflammatory cells stained with Runx-1 (I) surrounding eccrine glands. **(B)** Image (40X magnification) showing a zone marked in **(A)** showing positive Runx-1 stained cells (macrophages, M). **(C)** Dermic inflammatory infiltrated sample (I) stained with Runx-1. **(D)** Non-leprosy dermic sample (Non-LP) negative for Runx-1. **(E)** Comparison of the Runx-1 stained cells in leprosy vs. non-leprosy skin samples (*P* < 0.05). **(F)** Image (10x magnification) showing a leprosy skin sample (LP) stained with CD68 to confirm the presence of macrophages (M) in the inflammatory infiltrate (I) surrounding eccrine glands. **(G)** Image (40X magnification) showing the zone marked in **(F)** showing macrophages (M). All the figures have a 50-μm scale bar.

We established that Runx-1 transcription factor is not expressed in the dermal nerves of leprosy patients (Figure 2). On the other hand, immunostaining showed that the overexpression of Runx-1 in leprosy patients was due to its expression in the cells present in the dermal inflammatory infiltrate. The dermis showed a median of 98.5 cells stained for Runx-1 per field observed in leprosy patients (interquartile range (IQR) = 44.7–177 cells per field), while the median was only 4 cells per field in non-leprosy patients (IQR= 1.5–5.5 cells per field) (*p* < 0.0001) (Figures 3A–E). Later, we established using CD68 marker that the cells stained for Runx-1 correspond to macrophages that are generally distributed diffusely at the cutaneous layer level (Figures 3F–G).

**Figure 4:**
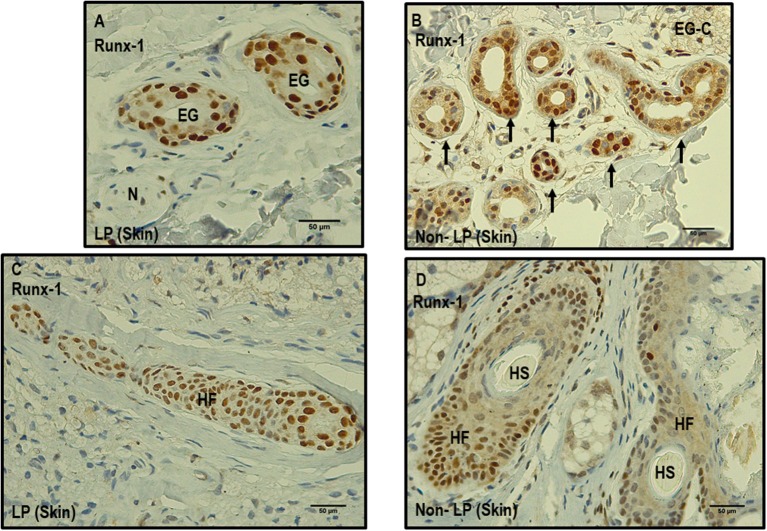
Comparison between leprosy skin and non-leprosy skin using immunohistochemistry with Runx-1 in eccrine glands and hair follicles. **(A)** Two eccrine glands in leprosy sample (LP) positive for Runx-1; N = nerve. **(B)** Eccrine gland conglomerate (EG-C) in a non-leprosy sample (non-LP) positive for Runx-1. **(C)** Hair follicle (HF) in a leprosy sample (LP) positive for Runx-1 **(D)**. Two hair follicles and two hair shafts (HF) in a non-leprosy sample (non-LP) positive for Runx-1. All images have a 50-μm scale bar.

Other skin structures in which Runx-1 marking was observed were the skin annexes (eccrine glands, hair follicles). At the level of the eccrine glands, a percentage of Runx-1-positive cells greater than 75% was observed in 63.3% of the eccrine structures of leprosy samples and in 73.3% of those of non-leprosy samples, a non-significant difference (*p* > 0.05). In the visualized hair follicles, the percentage of Runx-1-positive cells was greater than 75% in 50% of the hair follicles of leprosy samples and in 75% of non-leprosy samples, a slight but non-significant (*p* > 0.05) reduction in leprosy samples (Table 4, Figure 4).

**Table 4:** Differences in the expression of Runx-1 in eccrine glands and hair follicle between leprosy skin and non-leprosy skin.

**Sample**		**Leprosy patients (Skin)**	**Non-leprosy (Skin)**	**Chi-square *p*-value**
		***n* = 30 (%)**	***n* = 15 (%)**	
Stained cells in eccrine glands % (Runx-1)	<1%	2 (6.6%)	1 (6.6)	0.648
	1-25%	3 (10%)	0	
	25-75%	6 (20%)	3 (20%)	
	> 75%	19 (63.3%)	11 (73.3%)	
		***n*** **=** **22 (%)**	***n*** **=** **12 (%)**	
Stained cells in hair follicle % (Runx-1)	<1%	1 (4.5%)	0	0.480
	1-25%	1 (4.5%)	0	
	25-75%	9 (41%)	3 (25%)	
	> 75%	11 (50%)	9 (75%)	

### Identification of Hes-1 Expression in Cutaneous Structures of Leprosy Patients and Non-leprosy Individuals

**Figure 5:**
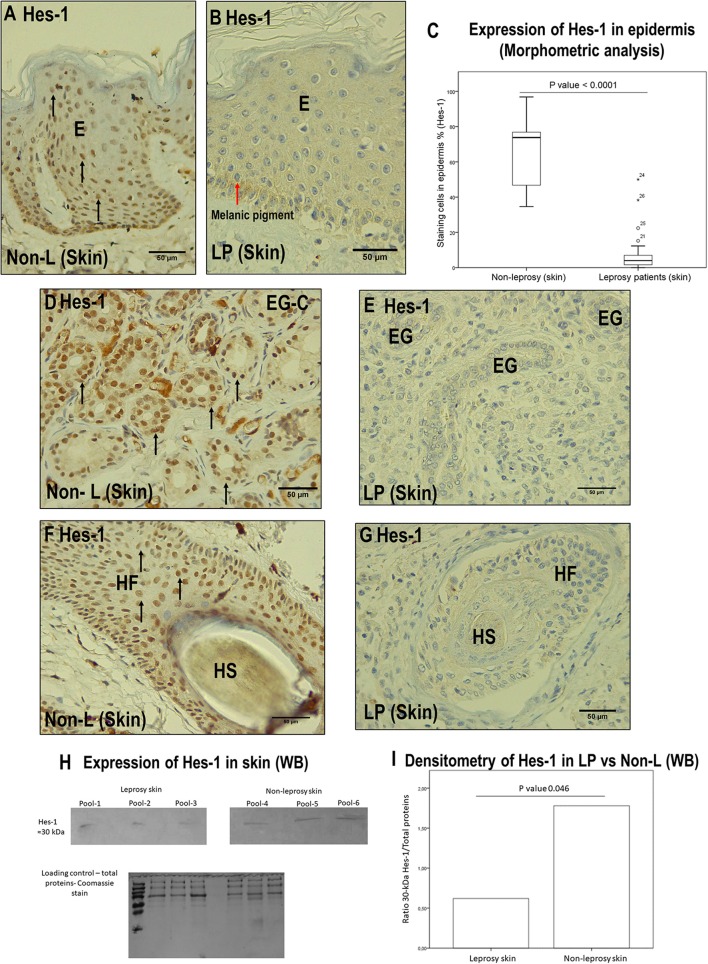
Comparison between leprosy skin and non-leprosy skin using immunohistochemistry with Hes-1 in the epidermis, eccrine glands, and hair follicles. **(A)** Epidermis (E) of a non-leprosy sample (non-LP) positive for Hes-1 in the Malpighian layer. **(B)** Epidermis (E) of a leprosy sample (LP) negative for Hes-1. The red arrow shows melanic pigment. **(C)** Whisker box chart showing a lower expression of Hes-1 in the epidermis of leprosy patients vs. non-leprosy patients, *p* < 0.0001. **(D)** Conglomerate of eccrine glands (EG-C) in a non-leprosy sample (non-LP) positive for Hes-1. **(E)** Three eccrine glands (EG) in a leprosy sample (LP) negative for Hes-1. **(F)** Hair follicle (HF) and hair shaft (HS) in a non-leprosy sample (non-LP) positive for Hes-1. **(G)** Hair follicle (HF) and hair shaft (HS) in a leprosy sample (LP) negative for Hes-1. **(H)** Western blot showing the bands for Hes-1 in leprosy skin, non-leprosy skin, and loading control (total proteins stained with Coomassie). **(I)** Densitometry showing a lower expression of Hes-1 in leprosy patients vs. non-leprosy patients, *p* < 0.05. All images have a 50-μm scale bar.

IHC of Hes-1 confirmed changes in expression in three skin structures of leprosy patients: epidermis, eccrine glands, and hair follicles. In the epidermis, a median of 4.2% of Hes-1-marked cells was observed in leprosy samples (IQR = 1.5–7.1 stained cells per field), while in non-leprosy samples it was 73.8% (IQR = 45.6–77 stained cells per field) (*p* < 0.0001) (Figures 5A–C).

At the level of the skin annexes, we also showed differences in the expression of Hes-1 when comparing leprosy and non-leprosy samples. In the eccrine glands, we observed that in 80% of leprosy samples, the percentage of Hes-1-positive cells ranged from <1% to 25%, while in 93.4% of non-leprosy samples, the percentage of Hes-1-positive cells was greater than 75% (*p* < 0.0001) (Figures 5D,E, Table 5). With respect to hair follicles, 83.3% of the follicles visualized in leprosy samples showed a percentage of Hes-1-positive cells ranging from <1% to 25%, while in 100% of the follicles evaluated in non-leprosy samples the percentage was greater than 75% (*p* < 0.0001) (Figures 5F,G, Table 5).

**Table 5:** Differences in the expression of Hes-1 in eccrine glands and hair follicle between leprosy skin and non-leprosy skin.

**Sample**		**Leprosy patients (Skin)**	**Non-leprosy (Skin)**	**Chi-square *p*-value**
		***n* = 30 (%)**	***n* = 15 (%)**	
Stained cells in eccrine glands % (Hes-1)	<1%	10 (33.4%)	0	<0.0001
	1–25%	14 (46.6%)	0	
	25–75%	4 (13.4%)	0	
	> 75%	1 (3.3%)	14 (93.4%)	
	Not observed	1 (3.3%)	1 (6:6%)	
		***n*** **=** **18 (%)**	***n*** **=** **14 (%)**	
Stained cells in hair follicle % (Hes-1)	<1%	7 (38.9%)	0	<0.0001
	1-25%	8 (44.4%)	0	
	25-75%	3 (11.1%)	0	
	> 75%	0	14 (100%)	

On the other hand, the IHC analysis of Hes-1 allowed us to rule out that this transcription factor is being expressed in the dermal nerve fibers of leprosy patients (Figure 2A). Finally, the IHC findings of Hes-1 were validated through Western blot, showing a significant reduction in the expression of Hes-1 in the skin samples of leprosy patients (*p* < 0.05) (Figures 5H,I).

### Histopathological and Gene Findings That Might Explain the Changes in the Expression of Hes-1 (Multivariate Analysis)

After establishing the dermal structures in which changes in the expression of Hes-1 were observed (epidermis, hair follicle, and eccrine glands), we decided to evaluate how the inflammatory, trophic, neural, and genetic changes found in the tissue could explain or be related to the reduction of Hes-1. For this, we performed a multinomial logistic analysis relating to the percentage of Hes-1-positive cells in these structures with gene findings (*Jagged-1, Notch-1*) and histopathological findings (trophic changes, inflammatory changes, and involvement of dermal nerves) in which a difference was found between leprosy and non-leprosy samples (Table 3, Table S3).

When relating these findings to the percentage of Hes-1-positive cells, we observed that changes in the expression in the epidermis, eccrine glands, and hair follicles was mainly related to inflammation of the cutaneous tissue (*p* < 0.0001). In addition, at the epidermis, eccrine glands, and hair follicles, the reduction of Hes-1 was also related to a lower expression of the Jagged-1 ligand (*p* < 0.05) (Table 6).

**Table 6:** Multivariate analysis of the histopathological and molecular variables that could explain the changes observed in the expression of Hes-1.

**Variables**	**Epidermis**		**Eccrine glands**		**Hair follicle**	
	**Chi-square**	***p*-value**	**Chi-square**	***p*-value**	**Chi-square**	***p*-value**
Tissue inflammation, Atrophic changes, Dermal nerve damage, *Jagged-1, Notch-1*	**73.27**	**<0.0001**	**73.86**	**<0.0001**	**89**	**<0.0001**
**Inflammation**	**19.9**	**<0.0001**	**25.35**	**<0.0001**	**27.05**	**<0.0001**
Atrophic changes	7.8	0.05	0.425	0.98	4.83	0.305
Dermal nerve changes	10.8	0.095	5.4	0.66	10.86	0.209
*Jagged-1*	7.8	0.05	**14.18**	**0.007**	**9.8**	**0.044**
*Notch-1*	4.9	0.17	8.31	0.081	2.8	0.579

*The bold values are showing statistical significance*.

### Differences in Cyclin D1 Expression in Nerve Fibers of Leprosy Patients and Non-leprosy Individuals

When determining the lack of overexpression of Notch components in dermal nerve fibers in leprosy patients, it was feasible that the neural damage model was not related to cyclin D1 expression (8). We therefore decided to evaluate the expression of cyclin D1 in the dermal nerves of a subsample of 10 leprosy and five non-leprosy samples.

**Figure 6:**
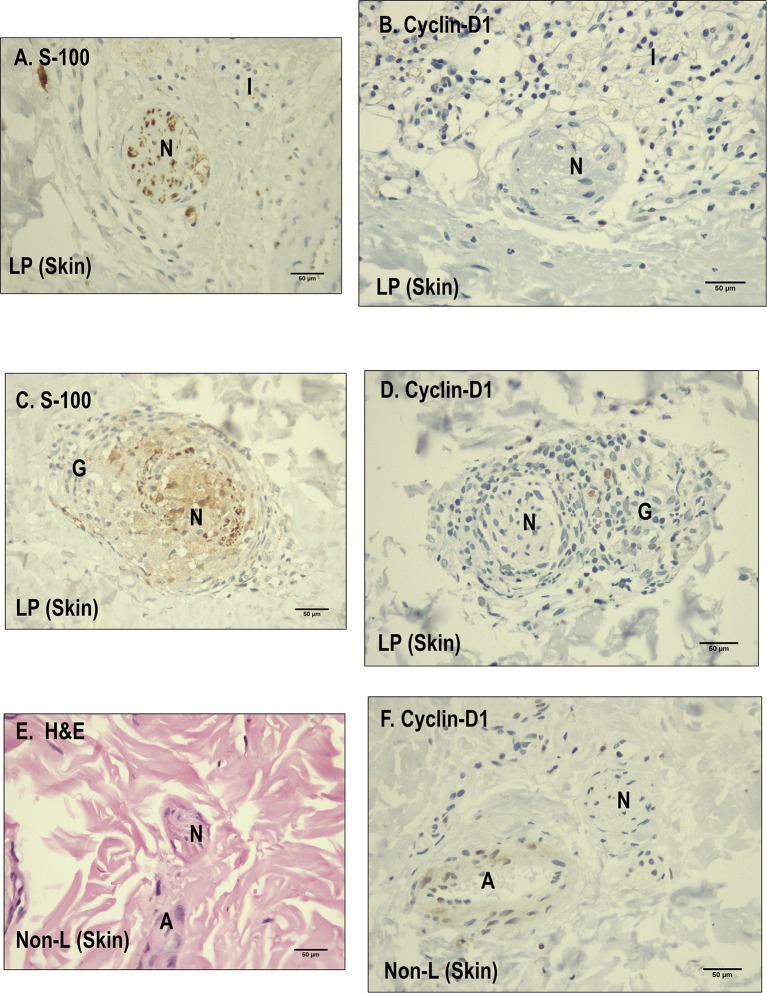
Comparison between leprosy skin and non-leprosy skin using immunohistochemistry with Cyclin D1 in dermal nerves. **(A)** Dermal nerve (N) stained with S-100 in a leprosy sample. **(B)** Dermal nerve (N) surrounded by inflammatory cells in a leprosy sample (LP) negative for Cyclin D1. **(C)** Dermal nerve surrounded by a granuloma in a leprosy sample (LP) stained with S-100. **(D)** Dermal nerve surrounded by a granuloma in a leprosy sample (LP) negative for cyclin D1. **(E)** Dermal nerve (N) stained with hematoxylin and eosin (H&E) in a non-leprosy sample (non-LP); A, Artery. **(F)** Dermal nerve (N) in a non-leprosy sample (non-LP) negative for cyclin-D1; A, artery.

With regard to the expression of cyclin D1 in the nerve fibers, 100% (*n* = 10) of leprosy samples showed no cyclin expression in the evaluated dermal nerves. Similarly, 100% (*n* = 5) of non-leprosy samples showed no expression of this cellular component in the nerves (Figure 6).

## Discussion

### Differences in the Expression of Some Components of the Notch Signaling Pathway Between the Cutaneous Samples of Leprosy Patients and Non-leprosy Individuals

When evaluating the transcription of genes related to the Notch signaling pathway, we found significant changes in the expression of *Hes-1* and *Runx-1* in leprosy samples, in fact, *Hes-1* gene was found to be downregulated in the samples of leprosy patients This allows us to infer that *Hes-1* would not be related to the deterioration of the dermal nerve fiber caused by *M. leprae*, given that *Hes-1* overexpression is necessary to induce nerve damage (10).

On the other hand, the reduction of *Hes-1* in the cutaneous samples of these patients makes us think that *M. leprae* could directly induce trophic alterations in the skin. This finding is related to what was reported by Lin et al. (20), who demonstrated that the reduction of Hes-1 compromises differentiation and cell proliferation in the epidermis and hair follicle, together with the pluripotential capacity of stem cells in the skin.

In addition, the compromise of *Hes-1* could be explained through results from this research, such as the reduction in the expression of the *Jagged-1* ligand and even the *Notch-1* receptor in skin affected by *M. leprae*. Since *Jagged-1* ligand–*Notch-1* receptor interaction does not occur, the cleavage and translocation of the Notch intracytoplasmic domain (NICD) at the nuclear level would be impaired, thus blocking the expression of *Hes-1* (6, 21).

The results of the increased transcription of *Runx-1* in the cutaneous samples of leprosy patients allow us to consider two hypotheses in this respect. The first is that the overexpression of this transcription factor occurs in the dermal nerve fiber, which could lead to its deterioration (10). Our second hypothesis is that the increase in the transcription of *Runx-1* is a consequence of its canonical expression in immune cells such as macrophages (22, 23), cells that have a marked activity *in situ* against *M. leprae* in the skin of MB patients (24).

With respect to *Hey-1*, no significant changes in its expression were observed, although it has been related to the genes of the *Hes* family in the processes of proliferation and cellular differentiation at cutaneous and nervous levels (25, 26). It is possible that this finding is related to what was reported in murine keratinocytes by Blanpain et al. (27), who found that *Hey-1* expression is significantly reduced with respect to *Hes-1* from embryonic day 17, and this would be related to an increased activity of Hes-1 in cell proliferation processes at the epidermal level.

Among the genes evaluated, we also expected to find an increase in *Numb* transcription, which is a Notch inhibitory membrane protein with the ability to bind and prevent cleavage and nuclear translocation of the NICD, thus blocking the transcriptional machinery of this signaling pathway (28). Therefore, in the absence of a difference in *Numb* expression between leprosy and non-leprosy samples, we rule out that the changes in *Notch* expression found in the skin of these patients are mediated by changes in the expression of this inhibitory protein.

Based on these findings, we proposed that changes in the expression of *Hes-1* in the skin of these patients could compromise the proliferation, differentiation, and immune response of dermal cells against this microorganism, and at this point in the research, we did not rule out that *Runx-1* expression was occurring in the dermal nerve fiber. It is for this reason that the immunohistochemical evaluation of Hes-1 and Runx-1 became necessary so as to establish the dermal structures in which the changes in the expression of these genes were taking place.

### Cutaneous Structures in Which There Are Differences in the Expression of Runx-1 in Leprosy Patients and Non-leprosy Individuals

IHC tests corroborated that the increase of Runx-1 in leprosy patients is linked to the presence of macrophages marked with CD68 in the inflammatory infiltrate. This finding would be related to the fact that Runx-1 is key to the maintenance and survival of these cells by inhibiting pro-apoptotic molecules such as Fas receptor and BH3-only protein Bim (22). On the other hand, the non-expression of Runx-1 in the dermal nerve fiber demonstrates that it is likely that this transcription factor is not a trigger for demyelination and nerve fiber damage.

As for the expression of Runx-1 at the level of the eccrine glands and hair follicles, to date, it is known that Runx-1 is a transcription factor that has been related to promoting stem cell activity at the level of the hair follicle (29, 30). In addition, this work could establish that Runx-1 is also expressed in the epithelium of the eccrine glands and that its action could be related to promoting the processes of stem cell proliferation and differentiation located on this cutaneous structure.

These findings exclude that the transcription factor Runx-1 is a trigger in the damage of the dermal nerve fiber or is involved with tissue changes in the skin of leprosy patients.

### Cutaneous Structures in Which There Are Differences in the Expression of Hes-1 in Leprosy Patients and Non-leprosy Individuals

The IHC evaluation allowed us to establish that the reduction in the expression of Hes-1 in the skin of leprosy patients is limited to three skin structures: epidermis, eccrine glands, and hair follicles. This is in comparison with non-leprosy individuals, in whom Hes-1 was expressed constitutively in these structures, according to Cleaton et al. (31).

**Figure 7:**
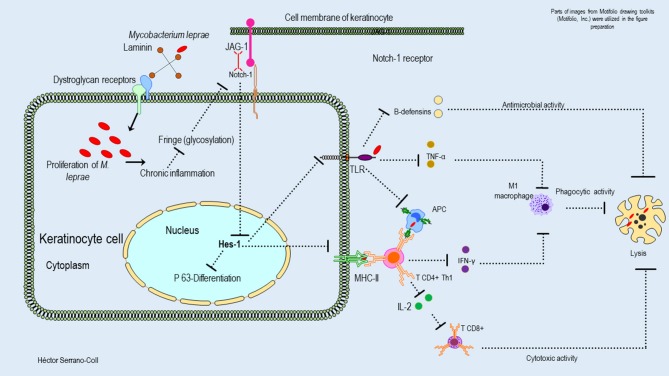
Hypothetical model of the possible mechanisms used by *M. leprae* to modulate the expression of the Notch signaling pathway in the epidermis and their consequences. This figure shows how *M. leprae* could be related to the basement lamina (laminin receptor–dystroglycan complex) of the keratinocytes. The proliferation of *M. leprae* inside of these cells could induce a chronic inflammatory process that would reduce the glycosylation of the Notch receptor and its interaction with the ligand Jagged-1. This fact could induce aberrant signaling and reduced expression of Hes-1, affecting the activation of the protein P-63, the differentiation of the keratinocytes, and the innate and adaptative immune response against *M. leprae*.

The reduced expression of Hes-1 in the epidermis of leprosy patients at the keratinocyte level would have a direct effect on the mechanisms of proliferation and differentiation in this cell (25). It is known that Hes-1 can directly induce the proliferation of keratinocytes by promoting the progression of the cell cycle at the level of the basal layer of the epidermis (6). It also facilitates the expression of the protein P-63, which is a key regulator in the development and differentiation of this cell-type, by inducing the expression of cytokeratins such as K1 and K10 (32, 33). Therefore, trophic changes in the epidermis of leprosy patients could be explained by *M. leprae*-induced neural damage and changes in Hes-1 expression. In addition, it is likely that changes in the expression of Hes-1 and Notch ligands and receptors will compromise the expression of Toll-like receptors (TLR) and major histocompatibility complex class II (MHC-II) molecules (34). Therefore, this cell de-differentiated would alter the innate immune response, the antimicrobial activity, the antigenic presentation, and the differentiation of CD4+ LT toward a Th1 and Th17 pattern, which are key in the activation of cells with cytotoxic and phagocytic activity against *M. leprae*. Figure 7 shows a hypothetical model of the possible mechanism used to *M. leprae* to modulate the Notch signaling pathway in the epidermis.

**Figure 8:**
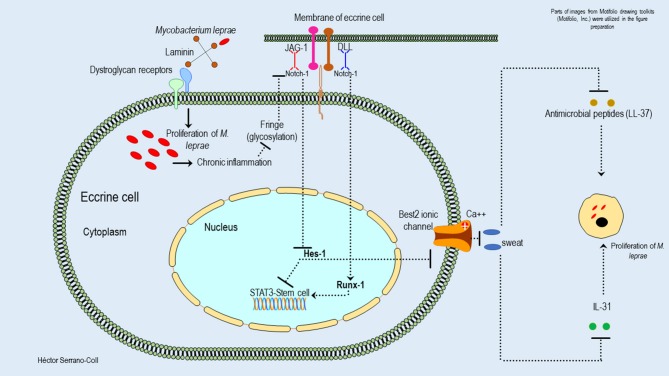
Hypothetical model of the possible mechanisms used by *M. leprae* to modulate the expression of the Notch signaling pathway in eccrine cells and their consequences. This figure shows how *M. leprae* could reduce the expression of Hes-1 in the eccrine cells. This would block the activation of STAT3 and the function of the stem cells located in this cutaneous annex. This effect could be partially mitigated by Runx-1. In addition, the reduction of the expression of Hes-1 could be associated with the reduction of sweat secretion, probably because the inhibition of the expression of the Best-2 channels, with a consequent reduction of secretion of antimicrobial peptides (cathelicidin LL-37) and cytokines (IL-31), could promote the proliferation of *M*. *leprae* and the interaction host-pathogen.

**Figure 9:**
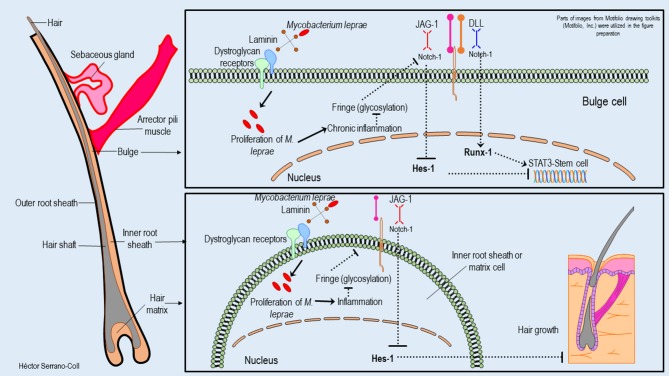
Hypothetical model of the possible mechanisms used by *M. leprae* to modulate the expression of the Notch signaling pathway in the hair follicle and their consequences. This figure shows how *M. leprae* could reduce the expression of Hes-1 in the hair follicle. This fact would block the activation of STAT3 and the function of stem cells located in the bulge, which is partially mitigated for the expression of Runx-1. In addition, the reduction of the Hes-1 expression could induce the loss of hair follicles in the skin of leprosy patients.

Changes in the expression of Hes-1 in the skin annexes (eccrine glands and hair follicles) of leprosy patients could compromise the pluripotential activity of stem cells located in the eccrine glands and in the promontory of the hair follicle (35, 36), considering that Hes-1 is key in the activation of STAT3 (Signal Transducer and Activator of Transcription 3), which is a transcription factor involved in the maintenance and differentiation processes of stem cells (37). Figures 8, 9 show a hypothetical model of the possible mechanism used to *M. leprae* to modulate the Notch signaling pathway in eccrine glands and hair follicles, respectively.

These findings allow us to establish, that alterations in the processes of reepithelialization and scarring in the skin lesions of leprosy patients could be related both to tissue compromise generated by leprosy neuropathy (38) and to changes in Hes-1 expression. In addition, we consider that in the future, modulation of this transcription factor could be a therapeutic alternative in the management of leprosy ulcers.

In the eccrine glands, it is also important to mention that the reduction of Hes-1 would compromise the secretory function of the luminal cells, which is mediated by ionic channels dependent on calcium, called Bestrophin-2 (Best2) (39). These channels have been extensively studied at the intestinal level, showing that the secretory activity of Best2 is dependent on the Notch signaling pathway and specifically on the expression of Hes-1 (40). Therefore, changes in its expression would reduce sweating, its antimicrobial activity (cathelicidin LL-37), and the expression of cytokines such as IL-31—key in the activation of keratinocytes (39)—in the cutaneous areas affected by *M. leprae*, which would facilitate the proliferation of this mycobacterium (Figure 8).

Another relevant finding of this research is that we did not find expression of Hes-1 in the nerve fibers of leprosy patients, which makes it unlikely that Hes-1 is involved in the deterioration of dermal nerve fibers (10).

### Histopathological and Gene Changes That Might Explain the Reduction in Hes-1 Expression in the Skin of Leprosy Patients

The main histopathological finding of this research associated with the reduction in the expression of Hes-1 in the skin of leprosy patients is the inflammation found at the level of the dermis, skin annexes, and subcutaneous cellular tissue. This could be related to the findings of Derada et al. (41), who observed that inflammatory processes at the cellular level reduce the expression of a glucosyltransferase such as “Fringe,” which has the function of adding residues of O-fucose to N-acetylglucosamine repetitions, located in the extracellular domain of the Notch receptor (NECD). The interaction of the ligand Jagged-1-receptor Notch-1, which activates this signaling pathway, depends on this glycosylation (42). Therefore, we propose that inflammation induced by *M. leprae* in the skin could cause alterations in the glycosylation of NECD, causing aberrant signaling of Notch and changes in the expression of Hes-1 (Figure 7).

In the epidermis, eccrine glands, and hair follicle another finding is that the expression of Hes-1 could be related to changes in the gene expression of the *Jagged-1* ligand since the reduced expression of this cellular component would limit the ligand-receptor interaction and would reduce the expression of Hes-1 (6, 21), as discussed previously.

On the other hand, based on the inflammatory mechanism explaining the reduction of Hes-1, we ask the question: why was the transcriptional factor Runx-1 not altered in leprosy patients, considering that some Notch transcription factors are affected in inflammatory environments? The answer we suggest is that Runx-1 can be expressed through two pathways, one that is given by the interaction of Jagged ligands with the Notch-1 receptor and a second that is mediated by the union of Delta-type ligands with this receptor (43); it has been demonstrated that this interaction (Delta-Notch) is not involved in inflammatory processes (41), which confirms our findings.

### Cyclin D1 Expression in Dermal Nerves of Leprosy Patients and Non-leprosy Individuals

Since there is no evidence of cyclin D1 expression in the dermal nerve fibers of leprosy patients, we rule out the possibility that neural damage in these patients has related to cyclin D1 expression or to the transcription factors of the Notch signaling pathway that were evaluated. It is probable that in these patients, dermal nerve fiber damage is linked to the reduction of transcription factors Oct-6 and Sox-10, which are positive regulators of the genes in charge of myelin maintenance in the nerve (unpublished data).

The absence of cyclin D1 in the dermal nerve fibers affected by *M. leprae* would be related to the lack of expression of transcription factors Hes-1 and Runx-1. Given that, if there is no cyclin D1 overexpression, it is unlikely that the expression of Numb—which is a Notch inhibitory protein—will be reduced, thus limiting cleavage and translocation at the nuclear level of the NICD, thus making the expression of Notch-associated transcription factors, which behave as a myelin repressor in the nerve, unfeasible (10).

## Conclusions and Future Perspective

The Notch signaling pathway is a cellular component that is not involved in the deterioration of the dermal nerve fiber but is related to some *M. leprae*-induced tissue changes in the skin of its host (epidermis, eccrine glands, and hair follicles). Such changes would be linked to a reduced expression of the transcription factor Hes-1, which then alters the processes of proliferation and differentiation in keratinocytes, eccrine luminal cells, and cutaneous stem cells, which would allow *M. leprae* to survive and proliferate in this tissue.

Around this infection and its relationship with the Notch signaling pathway, we still need to resolve some questions, such as:

1. What other components of the Notch signaling pathway could be involved in *M. leprae*-induced tissue and cell damage?
2. Could Runx-1 expression be a compensatory mechanism in the skin of leprosy patients, useful for coping with the tissue changes induced by the reduction in Hes-1?
3. How can we intervene in the modulation of Notch activity and restore Hes-1 expression in the skin of leprosy patients?
4. Could Hes-1 be used as an auxiliary marker in the diagnosis of leprosy?

These questions indicate that there is still a long way to go in understanding the cellular mechanisms proposed here. In addition, it is likely that from these findings or others related to changes in cell signaling induced by *M. leprae*, new and better tools will be developed in the future to facilitate the early detection of leprosy and allow us to take a step forward in the elimination of this disease.

## Limitations

This research was focused on MB patients because the aim of this study was to understand a mechanism of direct tissue damage related to the ability of *M. leprae* to modulate a signaling pathway such as Notch. Therefore, in the short term, it is necessary to study this mechanism in PB patients and to show whether changes of the Notch signaling pathway are present in this clinical spectrum.

## Data Availability Statement

The raw data supporting the conclusions of this article will be made available by the authors, without undue reservation, to any qualified researcher.

## Ethics Statement

This research was conducted in accordance with the international ethical standards given by the World Health Organization and the Pan American Health Organization, supported by the Declaration of Helsinki, promulgated in 1964, and the statutes given at the national level by resolution number 008430 of 1993 of the Ministry of Health of Colombia, which regulates health studies. In addition, it was endorsed by the institutional ethics committee for human research of CES University (act No 101 of March 3, 2017), and respective endorsement was obtained from the ethics committee or the legal representative of the institutions that participated in this project.

## Author Contributions

HS-C and NC-C designed the study, standardized the qRT-PCR and WB assays, and wrote the manuscript. Participants in this study were evaluated by HS-C. HS-C, JO, and LS-P standardized the IHC tests. JO interpreted the study's histopathological and immunohistochemical findings. HS-C performed the data analysis. JO and LS-P conducted a critical review. This research was directed by NC-C. All authors read and approved the manuscript.

### Conflict of Interest

The authors declare that the research was conducted in the absence of any commercial or financial relationships that could be construed as a potential conflict of interest. The handling editor declared a past co-authorship with two of the authors HS-C and NC-C.
