# Supplementary material for: Notch Signaling Pathway Expression in the Skin of Leprosy Patients: Association With Skin and Neural Damage
Source: Front Immunol. 2020 Mar 19;11:368. doi: 10.3389/fimmu.2020.00368 (PMC7096478; doi:10.3389/fimmu.2020.00368)
Supplement: Supplementary file 1 [file Table_1.DOCX]

**Supplementary material**

**Table S1.** **Antibodies used for immunohistochemistry analysis**

| **Target** | **Antibody** | **Clone** | **Isotype** | **Immunogen** | **Dilution** | **Positive control** | **Source** |
| --- | --- | --- | --- | --- | --- | --- | --- |
| Hes-1 | Anti-Hes-1 (mouse) | Monoclonal | IgG1 | Recombinant full-length Human Hes1 produced in HEK293T cells | IHC-P: 1:100 | thyroid | Abcam, ab 119776 |
|  | Reacts with: human |  |  |  |  |  |  |
| Runx-1 | Anti-Runx-1 (rabbit).  Reacts with: Human, mouse | Polyclonal | IgG | Synthetic peptide conjugated to KLH derived from within residues 400 to the C-terminus of Human Runx-1 | IHC-P: 1:250 | Thymus | Abcam, ab 35962 |
| S-100 | Anti-S-100 (rabbit).  Reacts with: human, mouse, rat, zebrafish, sheep. | Polyclonal | IgG | S100 isolated from cow brain. | IHC-P: 1:5600 | amygdala | Dako, Z 0311 |
| CD68 | Anti }-CD68  (mouse), clone (PG-M1)  Reacts with: human | Monoclonal | IgG3 | Human mononuclear spleen cell preparation containing more than 80% of Gaucher's cells | IHC-P: 1:600 | amygdala | Dako, M 0876 |
| Cyclin D1 | Anti-Cyclin D1 (rabbit).  Reacts with human | Monoclonal | IgG | Synthetic peptide derived from C-terminal segment of cyclin D1 | Non-apply | Mantle cell lymphoma | Master diagnostica, EP12 |

IHC-P: immunohistochemistry -paraffin

**Table S2. Expression of Runx-1 in inflammatory cells in dermis between leprosy skin and non-leprosy skin (visual scale)**

| **Sample** | | **Non-Leprosy (Skin)** | **Leprosy patients (Skin)** | **Chi-square P-value** |
| --- | --- | --- | --- | --- |
|  |  | **n=15 (%)** | **n=30 (%)** |  |
| inflammatory cells in dermis stain with Runx-1 | Yes | 0 | 28 (93.3%) | <0.0001 |
|  |  |  |  |  |
|  | No | 15 (100%) | 2 (6.7%) |  |
|  |  |  |  |  |

**Table S3. Differences in the expression of Hes-1 in epidermis between leprosy skin and non-leprosy skin (visual scale)**

| **Sample** | | **Non-Leprosy (Skin)**  **n=15 (%)** | **Leprosy patients (Skin)**  **n=30 (%)** |  | **Chi-square P-value** |
| --- | --- | --- | --- | --- | --- |
| Staining cells in epidermis % (Hes-1) | < 1% | 0 | 18 (60%) |  | <0.0001 |
|  | 1-25% | 0 | 8 (26.6%) |  |  |
|  | 25-75% | 3 (20%) | 2 (6.7%) |  |  |
|  | > 75% | 12 (80%) | 2 (6.7%) |  |  |

**Table S4. Description of pathological patterns in dermal nerves for immunohistochemistry S-100**

| **Immunohistochemistry S-100** | | |
| --- | --- | --- |
| **Characteristic of dermal nerves** | **Interpretation** | **Reference** |
| Intact | Dark staining, large fibrillar structures in a wavy pattern without inflammatory cells inside | Dhakhwa R, Acharya S, Pradhan S, Shrestha SB, Itoh T. Role of S-100 Immunostain as An Auxiliary Diagnostic Aid in Leprosy. JNMA J Nepal Med Assoc. 2017;56(205):141-4 |
| Infiltrated | Dark staining, fibrillar structures in a wavy pattern associated with inflammatory cells |  |
| fragmented | Staining of small structures inside of a granuloma |  |
| Absent-destroyed nerves | No dark staining fibrillar structures within or outside the granuloma in an adequate biopsy containing subcutaneous fat and/ or multiple granulomas |  |

**Table S5. Sociodemographic and clinical characteristics of non-leprosy individuals.**

| **Characteristic of the individuals**  **(non-leprosy)** | | n=15 (%) |
| --- | --- | --- |
| **Sex** | **M** | 6 (40%) |
|  | **F** | 9 (60%) |
| **Average age* (median/range)** | | 42.8 (30-56) |
| **Geographic area** | **Antioquia** | 15 (100%) |
| **Personal or familiar background of leprosy** | **Yes** | 0 |
|  | **No** | 15 (100%) |

**Table S6. Differences histopathological between leprosy skin and non-leprosy skin**

| **Variable** | **Leprosy patients (skin)** | **Non-leprosy (skin)** | **Chi-square P-value** |
| --- | --- | --- | --- |
| **Inflammation** | 29 (96.6%) | 0 | <0.0001 |
| **Trophic changes** | 28 (93.3%) | 0 | 0.002 |
| **Dermal nerve changes** | 29 (96.6%) | 0 | <0.0001 |
