# Supplementary material for: Notch Signaling Pathway Expression in the Skin of Leprosy Patients: Association With Skin and Neural Damage
Source: Front Immunol. 2020 Mar 19;11:368. doi: 10.3389/fimmu.2020.00368 (PMC7096478; doi:10.3389/fimmu.2020.00368)
Supplement: Supplementary file 2 [file Image_1.pdf]

Supplementary Figure 1.

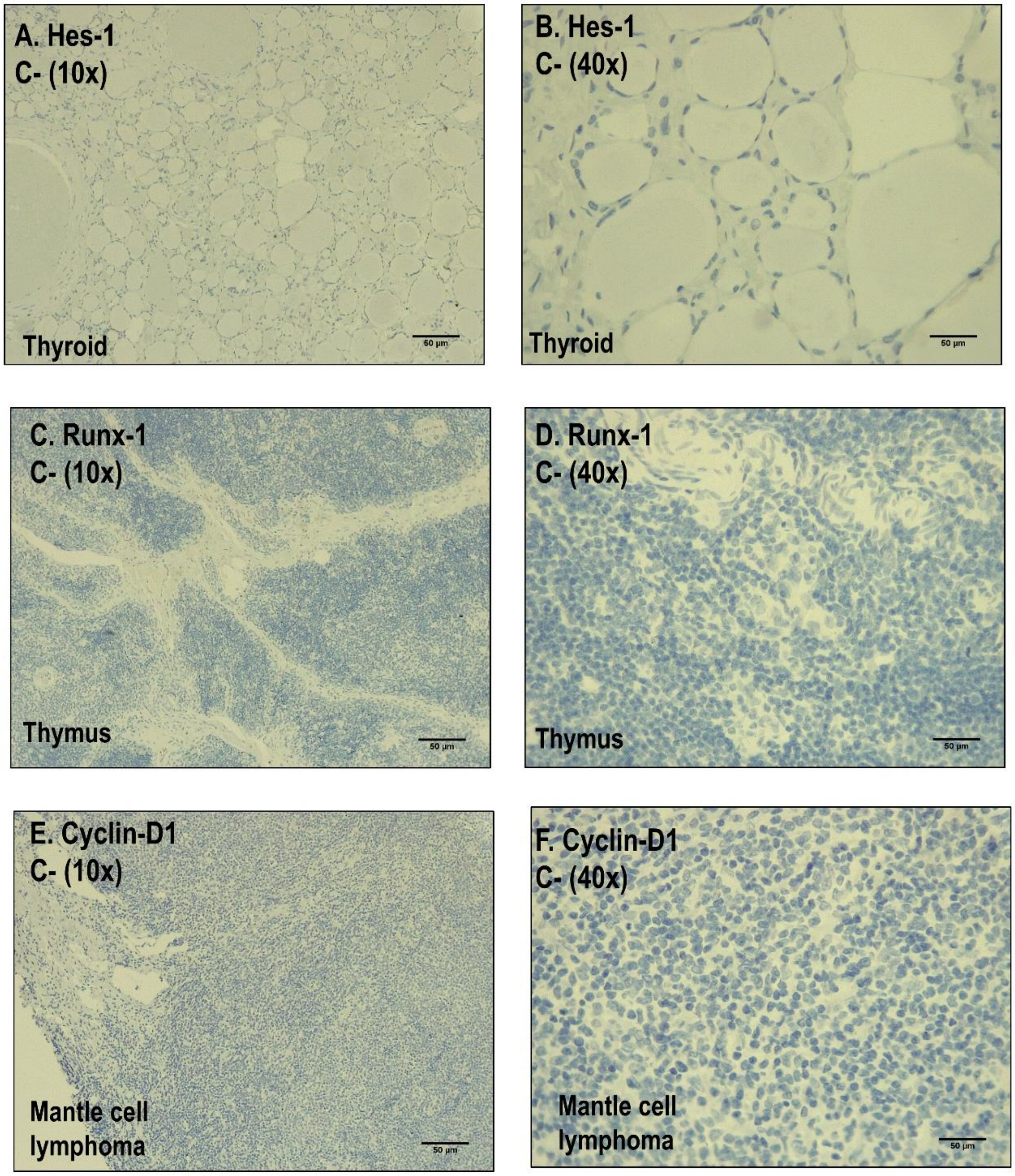

Supplementary Figure S1. Negative control used in immunohistochemistry to validate primary antibodies. (A) (Hes-1) This image (10x) shows non-stained nuclei in the thyroid follicular cells. **(B)** (Hes-1) This image (40x) confirms non-stained nuclei in the thyroid follicular cells. **(C)** (Runx-1) this image (10x) is a sample of thymic tissue, in which non-stained nuclei. (D) (Runx-1) This image (40x) shows non-stained nuclei in thymic cortex cells. **(E)** (cyclin D1) This image (10x) shows non-stained nuclei in a mantle cell lymphoma. **(F) (**cyclin D1) This image (40x) shows non-stained nuclei in neoplastic mantle cells. All images have a scale bar of 50 µm.

Supplementary Figure 2.

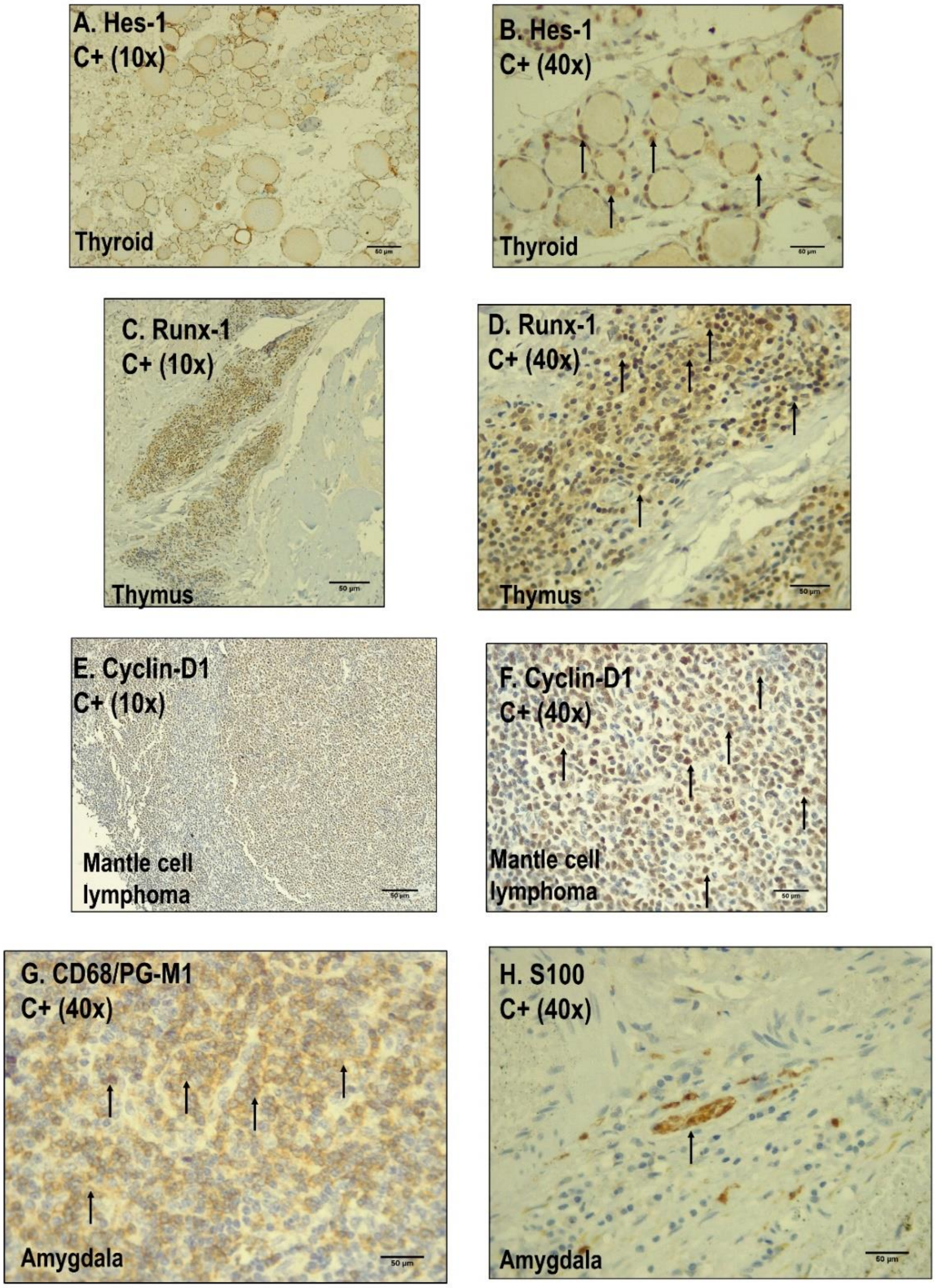

Supplementary Figure S2. Positive controls used in immunohistochemistry to validate primary antibodies. (A) This image (10x) shows Hes-1 staining in thyroid follicular cells. **(B)** This image (40x) shows stained nuclei with Hes-1 in thyroid follicular cells. **(C)** This image (10x) shows thymic tissue with Runx-1 staining. **(D)** This image (40x) shows stained nuclei with Runx-1 in thymic cortex cells. **(E)** This image (10x) shows cyclin D1 staining in mantle cell lymphoma. **(F)** This image (40x) shows stained nuclei with cyclin D1 in neoplastic mantle cells. **(G)** This image (40x) shows stained with CD68 (PG-M1) the cytoplasm and cell membrane of several macrophages in amygdala. **(H)** This image (40x) shows a nerve stained with S-100, and this staining is cytoplasmic and nuclear. All images have a scale bar of 50 µm. Arrows show the primary antibodies staining.

Supplementary Figure 3.

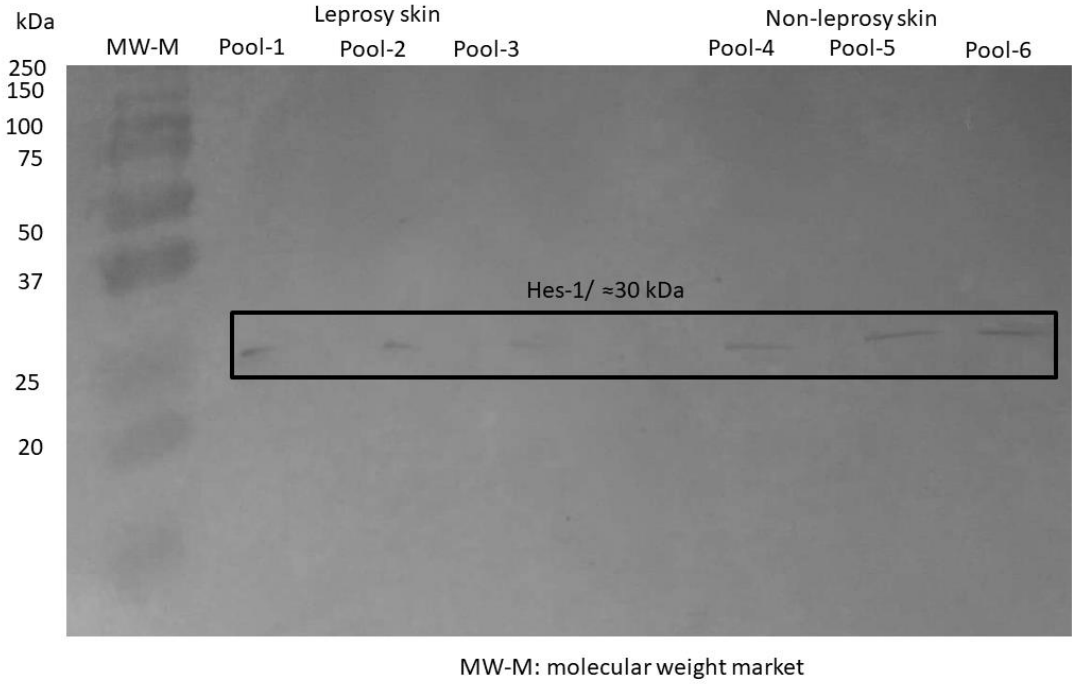

Supplementary Figure S3. Western blot to validate primary antibody (Hes-1), and this image shows expect bands of 30 kDa for Hes-1.

Supplementary Figure 4.

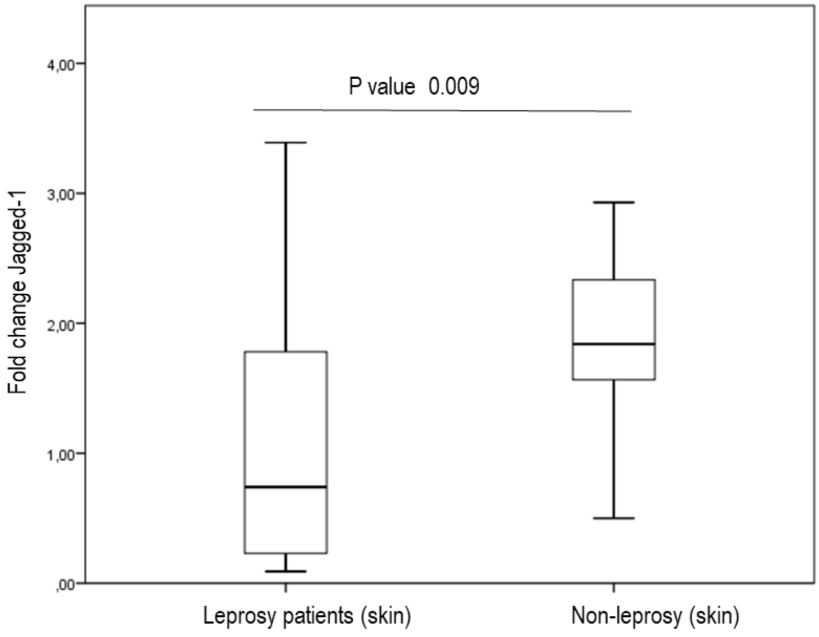

Supplementary Figure S4. This figure shows a lower gene expression of the Jagged-1 ligand in the skin of leprosy patients than in the skin (non-leprosy), and this difference is statistically significant.
