# Supplementary material for: Notch Signaling Pathway Expression in the Skin of Leprosy Patients: Association With Skin and Neural Damage
Source: Front Immunol. 2020 Mar 19;11:368. doi: 10.3389/fimmu.2020.00368 (PMC7096478; doi:10.3389/fimmu.2020.00368)
Supplement: Supplementary file 3 [file Data_Sheet_1.pdf]

Supplementary methods

H&E stain

Skin tissue sections of 4 µm were deparaffinized in an oven at 58°C overnight, followed by three 5-minute xylol immersions. After deparaffinization, the tissue was rehydrated through a series of decreasing concentrations of alcohols. Then, the tissue section was stained with hematoxylin for 6 minutes. The excess of dye was removed using a 2% acetic acid solution for 20 seconds and it was neutralized of the tissue with an immersion in water for 2 minutes. After, the tissue section was stained with eosin for 5 minutes and it was dehydrated using a series of alcohol solutions in ascending concentrating. Finally, the tissue section was clearing with two immersions in xylol.

Bacillary index (BI)

Stain slit skin smear samples and nasal swabs were stained with Zielh- Neelsen (ZN) to test for acid-alcohol-resistant bacilli. ZN staining was performed with a steam emission of fuchsine for 10 minutes, discoloration with acid-alcohol for 3 minutes and coloration of contrast with methylene blue for 2 minutes. In addition, BI was calculated using Ridley's logarithmic scale. Moreover, at the time for taking of bacilloscopy, four patients (13.3%) were new leprosy cases, 20 (66.7%) were in treatment, and six (20%) were post-treatment.

Measurement of antigen-specific serum antibodies (IgM anti NDO-LID)

Serum samples were collected for subsequent serologic evaluation. Briefly each well of 96-well ELISA plates (Nunc-Immuno 96-well, Polysorp plates) was coated with 1 µg/ml NDO-LID (Natural Octyl Disaccharide-Leprosy IDRI Diagnostic) antigen at room temperature then blocked using 100 µl blocking buffer (1% bovine serum albumin, BSA/ phosphate-buffered saline, PBS/ PBS + Tween20, PBS-T). Plates were incubated for 1 h with agitation at room temperature. Plates were washed (5 PBS-T + 2 PBS), and 50 µl serum (1:200 dilution in BSA 0.1%/ PBS/ PBS-T) was added, followed by a 1 h of incubation with agitation at room temperature. Subsequently, 50 µl horseradish peroxidase (HRP)-conjugated detector diluted in BSA 0.1%/ PBS/ PBS-T was added and plates incubated for 1 h with agitation at room temperature. Three detectors were evaluated: anti-human IgG, anti-human IgM and protein A (Rockland Immunochemicals Inc., Limerick, PA, USA). After incubation and

washing, 50 µl TMB substrate was added for 15 min in the dark at room temperature, then stopped by adding 25 µl 1 N sulfuric acid. Optical densities (OD) were measured at 450 nm using an ELISA plate reader (Spectrophotometer Bio-Rad Xmark). Cut-off values were assessed as the average OD plus two standard deviations obtained from serum ($n = 100$) of healthy individuals that resided in an area not endemic for leprosy. Cut-off values of 0.380 was obtained for IgM anti-NDO-LID**.**

Western-Blot (WB)

Western blot analysis was performed to validate the primary antibody related with Notch signaling pathway, and to make a quantitative evaluation of the expression of Hes-1 in a sub-sample of 10 leprosy patients and in 10 controls. This subsample was distributed in six pools: Pool-1: cutaneous samples 2, 9, 16 (leprosy patients); Pool-2: 48, 50, 8 (leprosy patients), Pool-3: 3, 10, 15, 17 (leprosy patients); Pool-4: 19, 25, 29 (non-leprosy), Pool-5: 23, 28, 34, 35 (non-leprosy), Pool-6: 19,37,44 (non-leprosy).

Protein extraction

These 20 skin samples were stored at -20 ° C, thawed at room temperature, macerated and 30 mg of skin were taken, which were centrifuged at 12,000 x g for 10 minutes at 4 ° C. Subsequently, 2 ml of lysis solution (1 ml of TRI Sigma-Aldrich reagent and 1 ml of C7559 chloroform, Sigma-Aldrich) was added to the tissue. Then, this mix was incubated for 25 minutes at room temperature and centrifuged at 12,000 x g for 15 minutes at 4 ° C to separate the proteins. After, the supernatant was transferred to a new tube, and 1200 µL of acetone was added and incubated for 4 hours. At the end of the incubation, the samples were centrifuged again at 12,000 x g for 15 min and the acetone was discarded. Then, 20 µL of reconstitution buffer was added. Finally, the protein concentration was determined using a Nanodrop 1000 spectrophotometer (Nanodrop Technologies, Wilmington, USA) and the samples were preserved at -20 ° C until western blotting.

Electrophoresis and WB

Equal protein concentrations (15 µg) were used in this analysis. Then, these samples were loaded on 8% polyacrylamide gel. Protein electrophoresis was carried out at 80 volts, for 30 minutes at 22°C, and then increased the voltage to 120 for 60 minutes at 22°C. After, proteins

were transferred to PVDF membranes (Bio-Rad, Hercules, CA, USA), using a wet electroblotting system (Mini Trans-Blot; Bio-Rad), and the membranes were blocked with non-fat milk at 5% in TBS with 0.1% tween-20 (1X TBST) for 60 minutes**.**

After this the proteins were transferred to PVDF membranes (Bio-Rad, Hercules, CA, USA), using a wet electroblotting system (Mini Trans-Blot; Bio-Rad) and the membranes were blocked with skim milk at 5% in TBS with 0.1% tween-20 (1X TBST) for 60 minutes**.** The membranes were incubated with mouse monoclonal anti-human Hes-1 antibody (1:1000) (Cat. No. ab119776, Abcam), to 4 °C overnight. Detection was performed using as secondary antibodies anti-mouse IgG peroxidase conjugated antibody produced in rabbit (Cat. No. A9044, Sigma-Aldrich®; St. Louis, MO, USA). As loading control, we used total protein extracts stained with Coomassie (Cat. No. 1610786, Bio-safe, Bio-Rad). All western-blot analysis were performed for duplicate.

The bands of interest for Hes-1 was visualized using an image analyzer (EpiChemi Dark Room, UVP, Upland, USA). Finally, digital images were analyzed using ImageJ 1.52 software, previously described in: Schneider CA, Rasband WS, Eliceiri KW. NIH Image to ImageJ: 25 years of image analysis. Nat Methods. 2012.
